# Drone Delivery of Insecticide Is Uneven Yet Sufficiently Controls Subterranean Weevils Infesting Sweet Potato Plants

**DOI:** 10.3390/plants14223511

**Published:** 2025-11-18

**Authors:** Koichiro Fukami, Kimiyasu Takahashi, Senlin Guan, Katsuya Ichinose

**Affiliations:** 1 Chikkugo Research Station, NARO Kyushu Okinawa Agricultural Research Center, Izumi 833-0041, Fukuoka, Japan; fukami.koichiro693@naro.go.jp (K.F.); takahashi.kimiyasu073@naro.go.jp (K.T.); guan.senlin291@naro.go.jp (S.G.); 2 Okinawa Office, NARO Kyushu Okinawa Agricultural Research Center, Itoman 901-0336, Okinawa, Japan; 3 Independent Researcher, Tsukuba 300-1252, Ibaraki, Japan

**Keywords:** *Cylas formicarius*, diamide insecticide, *Euscepes postfasciatus*, *Ipomoea batatas*, sweet potato weevil, unmanned aerial vehicle

## Abstract

Drone insecticide spraying is generating increasing concern and interest among academics and the public. However, the differences in the quantity of insecticide delivered by this method, and its efficacy in pest control for individual plants, remain to be evaluated. We examined the distribution and quantity of an insecticide sprayed on sweet potato plants and the method’s efficacy in controlling a subterranean weevil infestation. To evaluate delivery patterns, water-sensitive paper was placed on the canopy of the plants, and the insecticide was sprayed from a drone at the registered concentration. Although there was minimal deflection of flight paths (under crosswinds ≤ 3.0 m/s), the distribution varied across the field. The quantity administered was within the regulated range for the insecticide. Efficacy was not significantly influenced by either the quantity administered or pattern of spraying, and the drone application resulted in an equivalent level of control as a conventional ground-based application. While the quantity of the insecticide applied to the canopy was uneven, the method’s efficacy was satisfactory at the field scale. These findings can be used to develop safe and cost-effective methods for the drone application of pesticides.

## 1. Introduction

Reducing investments in cost and labor is becoming increasingly important in agriculture. Advances in drone technology have increased the efficiency of agricultural practices, particularly in the application of synthetic pesticides [1,2,3]. In recent decades, studies on pesticide application have demonstrated that pesticide efficacy is influenced by the mode of its dispersion onto plants [4,5]. However, few studies have examined patterns of insecticide delivery by drones or the control efficacy of insecticides relative to the quantity of insecticide applied. It remains unclear whether these traits differ among insecticides.

The selection of appropriate pesticides is essential for efficient pest control in agriculture, and reducing pesticide use is vital for protecting human health and the environment. To achieve this, pesticides must be sprayed precisely at targeted locations to prevent them from drifting into non-targeted areas [2]. Precise agriculture, which involves the target-specific application of the desired quantities of pesticides with appropriate control, has recently attracted attention, particularly with respect to the use of unmanned aerial vehicles (UAVs) in pest management [6,7]. Although aerial application is as effective as ground-based spraying [8], UAV application can be more practical and advantageous than ground-based application because UAVs are less affected by geographical conditions, thus reducing both the labor load and cost [9]. In evaluating such UAV use, it is necessary to examine the evidence linking the distribution of insecticide droplets to the insecticide’s efficacy, even at the level of individual plants.

The greatest advantage of using UAVs for pesticide application is that they allow for the precise targeting and control of the quantity of pesticide applied [2,7]. Along with the associated costs and labor savings, this flexibility in terms of pesticide delivery by UAVs has contributed to their increasing popularity. The precise delivery of pesticides depends on physical, chemical, and biological factors, such as the geographical properties of the field, the specifics of the drone used, the drone height and velocity, weather conditions, pesticide traits, the type of vegetation, and the crop growth stage [3,9,10,11]. Owing to the development of UAV technology, pesticides can now be delivered to precise locations and in the required quantities [12,13,14]. UAVs are thus useful for pest control via spot application, which is appropriate when the individual crop plants grow separately, such as in cereals and most vegetables. In contrast, pesticides’ distribution onto crops with intertwined vines requires the uniform delivery of pesticides, rather than spot application. If the vines of a plant are covered with those of other plants, it will receive less of the pesticide and will thus experience pest infestation. Sweet potatoes are a typical example of a crop with intertwined vines, making effective pest control difficult.

Here, sweet potatoes and two subterranean pest weevil species were chosen as pest management models for evaluating the drone-based application of pesticides. Although sweet potato crops have fewer insect pest species than other root crops, such as potato or radish [15], they suffer severe damage from the sweet potato weevil in the genus *Cylas*, including *C. formicarius* Fab. (Coleoptera: Apionidae), as well as from *Euscepes postfasciatus* (Fairmaire) (Coleoptera: Curculionidae) in tropical and subtropical regions [16,17]. These weevils have been identified as the most important insect pests in sweet potato farming on the Nansei Islands, Japan [18]. Their conventional control involves the application of insecticides such as organophosphates, pyrethroids, neonicotinoids, and carbamates [19,20]. However, with increasing public concern regarding the health and environmental effects of these insecticides, modern agricultural practices recommend avoiding their use. Diamides, which have recently been introduced into sweet potato cultivation, are likely to achieve better control of these pests than the previously used insecticides [21,22,23,24].

When insecticides are applied via spraying, they are transferred from the surface of the plant to the other parts, and they persist within the plant, exhibiting systemic delivery. As sweet potato weevils spend most of their lives underground within the sweet potato plant [25], they can consume insecticides that are present within the plant body. Systemic pesticides are thus useful for controlling pests on parts of the plant that other insecticides cannot reach directly and may therefore reduce pesticide use during the residual period [4,26]. Two new diamides, chlorantraniliprole and cyantraniliprole, are carried systemically throughout the plant after their application, and they exhibit control efficacy that is equivalent to that of conventional insecticides [27]. This study examined the variation in the quantity of an insecticide applied by a drone and its efficacy in controlling weevils in individual plants.

## 2. Results

### 2.1. Distribution of Insecticide Droplets

**Figure 1:**
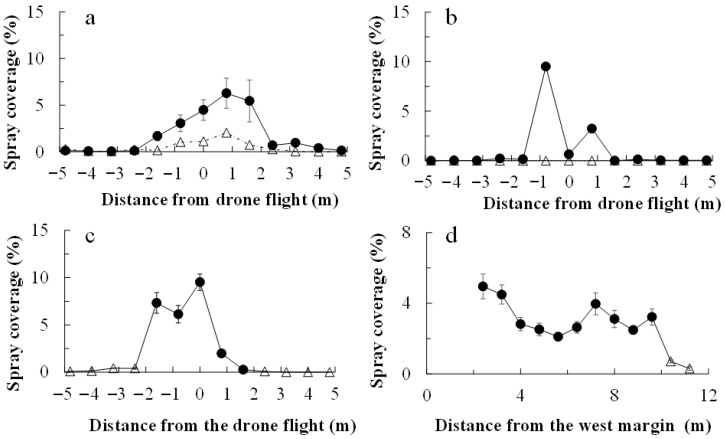
Chlorantraniliprole (**a**–**c**) and cyantraniliprole (**d**) droplet distribution following drone application. Droplets were detected on water-sensitive papers laid across the drone flight swath (0 m) in the drone application area of a sweet potato field. Chlorantraniliprole was applied on 21 August 2019 (**a**), 16 October (**b**), and 27 October (**c**) 2020, and cyantraniliprole was applied on 28 October 2020 (**d**). For chlorantraniliprole application, the drone flew along the 0 m path, with the swath extending on both sides of the flight path. For cyantraniliprole, it flew along lines located 0, 4, and 8 m from the western margin. The open triangles and closed circles represent papers placed off and within the drone swath, respectively.

**Figure 2:**
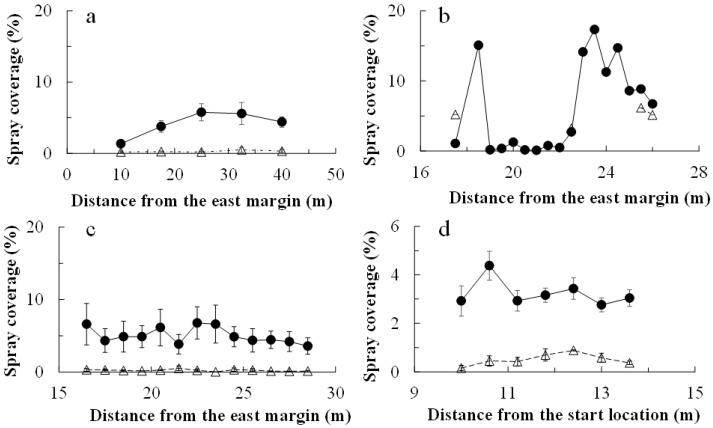
Chlorantraniliprole droplet coverage (mean ± se) following drone application. Droplets were detected on water-sensitive paper laid at specific distances from the eastern margin of the field (0 m; start of drone flight). Chlorantraniliprole was applied on 21 August 2019 (**a**), 16 October 2020 (**b**), and 27 October 2020 (**c**) and cyantraniliprole was applied on 28 October 2020 (**d**). For chlorantraniliprole, the drone flew along one flight line (0 m) in all applications. For cyantraniliprole, it flew along the 0, 4 and 8 m lines. The open triangles and closed circles represent papers placed off and within the drone swath, respectively.

In all applications, chlorantraniliprole and cyantraniliprole droplets were distributed much more on the water-sensitive papers within the drone swath (i.e., 2.0 m on either side of the flight line) than on those placed off the swath (Figure 1). The insecticide coverage was greater on the papers within the swath (Figure 2). The chlorantraniliprole coverage (as a percentage of paper stained) was similar for all three application dates. The cyantraniliprole coverage was approximately half that of chlorantraniliprole. The chlorantraniliprole coverage remained fairly constant with the increasing flight distance from the eastern margin (where the flight started) on 21 August 2019 and 27 October 2020, whereas on 16 October 2020, there was very low coverage at 19.0–22.0 m from the eastern margin; the coverage before and after this range was twice as high (Figure 2).

### 2.2. Quantity of Insecticide Applied

**Figure 3:**
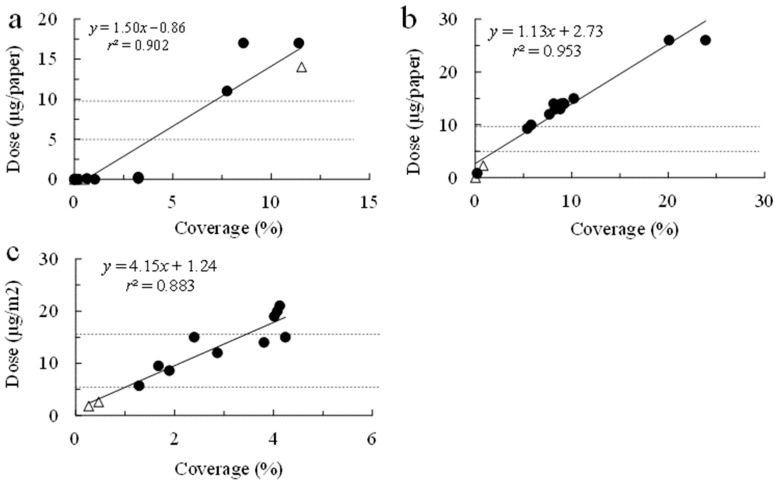
Quantities of insecticide deposited on the water-sensitive paper by drone for chlorantraniliprole on 16 October 2020 (**a**) and 27 October 2020 (**b**) and cyantraniliprole on 28 October 2020 (**c**). Open triangles and closed circles correspond to papers placed off and within the drone swath, respectively. Broken lines represent lower and upper limits of the registered insecticide quantities in Japan: 4.94 to 9.88 µg for chlorantraniliprole and 7.62 and 15.26 µg for cyantraniliprole.

The quantity of the insecticide applied was significantly linearly associated with the insecticide coverage (chlorantraniliprole, 16 and 27 October 2020, respectively: *t* = 10.910 [df = 14, *p* < 0.001] and *t* = 16.169 [df = 14, *p* < 0.001]; cyantraniliprole 28 October 2020: *t* = 8.681 [df = 11, *p* < 0.001]; Figure 3). For the cyantraniliprole applications on 16 and 27 October, the regression coefficients were comparable, at 1.50 and 1.13, respectively, while for the cyantraniliprole application, the coefficient was substantially higher, at 4.15. The quantity of insecticide deposited on the water-sensitive paper (i.e., in μg per paper) was estimated via regression for each application date (Figure 3). For all application dates, the means (±se) of the quantities of the insecticide deposited on the swath were within the ranges of the registered quantities: chlorantraniliprole—9.37 ± 1.21 μg (*n* = 49) and 8.40 ± 0.61 μg (*n* = 65) for first and second applications, respectively; cyantraniliprole application—14.7 ± 0.75 μg (*n* = 70). The quantities deposited on the off-swath papers were below the lower limit of the registered quantities for each insecticide: chlorantraniliprole, 4.04 ± 0.53 μg (*n* = 34) and 2.95 ± 0.05 μg (*n* = 32) for the first and second applications, respectively, and cyantraniliprole application, 3.35 ± 0.36 μg (*n* = 14).

**Figure 4:**
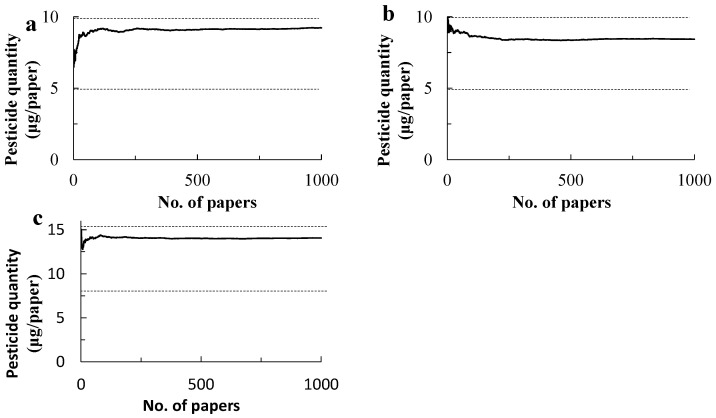
Estimated quantities of insecticide deposited on the water-sensitive papers placed under the drone flight swath on 16 October 2020 (**a**) and 27 October 2020 (**b**) and cyantraniliprole on 28 October 2020 (**c**). Paper collection was repeated one to one thousand times in each application, and the quantity per paper was calculated. Broken lines represent lower and upper limits of the registered insecticide quantities in Japan: 4.94 to 9.88 µg for chlorantraniliprole and 7.62 and 15.26 µg for cyantraniliprole.

The repeated collection of papers by bootstrapping methods showed that the per paper dose of chlorantraniliprole was asymptotically close to about 9 µg when the collection reached 150 to 200 papers, being within the registered quantity for the paper sizes from 4.94 to 9.88 µg (Figure 4). The papers amount to a 0.30 m^2^ and 0.40 m^2^ area, respectively, in both applications. In turn, the size makes a square of 0.54 to 0.63 cm in length or a circle with a 0.61 to 0.71 m diameter. Likewise, in the bootstrapping of cyantraniliprole, the per paper dose varied minimally after the collection of 150 to 200 papers, asymptotically reaching 14.7 µg, which was between the registered dose of 7.62 and 15.26, and again the area size for even distribution was estimated to be from 0.30 to 0.40 m^2^.

### 2.3. Occurrence of Weevils at Each Site

**Figure 5:**
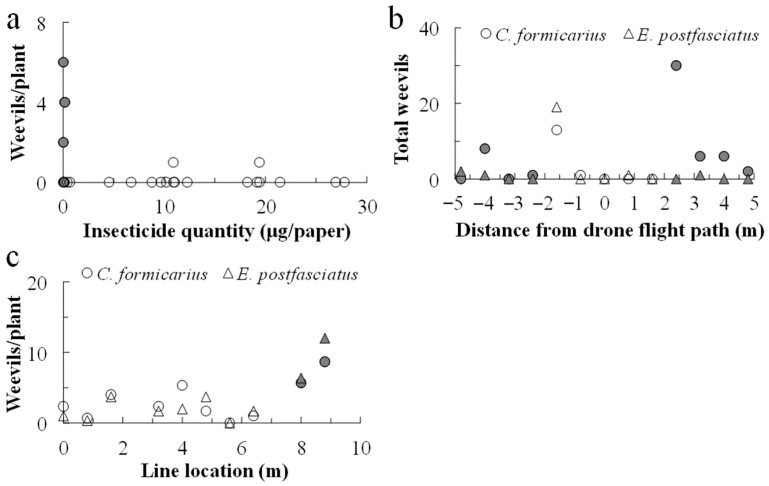
Numbers of weevils detected on the plants in the area targeted for drone spraying. For chlorantraniliprole application, the drone flew along the 0 m line; in 2019, only *C. formicarius* was collected (**a**), while in 2020 (**b**) both weevil species were collected. For the cyantraniliprole application in 2020 (**c**), the drone flew along lines 0, 4, and 8 m from the west margin, and plants were collected along lines from 0 to 8.8 m from the west margin at intervals of 0.8 m. For this experiment, it was assumed that the insecticides were deposited on the ground within 2.0 m on either side of the flight path. Closed symbols indicate plants off the flight swath.

At 26, 45, and 87 d after the 2019 chlorantraniliprole application, 296 *C. formicarius* and 7 *E. postfasciatus* weevils were collected at the three sampling sites. Owing to its low occurrence, *E. postfasciatus* was excluded from further analysis. Fewer weevils were collected from plants within the drone swath (2.0 m on either side of the flight path), with the majority collected outside the swath (Figure 5a). Significantly more weevils were collected off the swath (mean ± se, 34.5 ± 10.8 per location) than within the swath (4.0 ± 2.6; *F*_1,11_ = 6.247, *p* = 0.030).

Following the two chlorantraniliprole applications in 2020, 83 plants were collected, 34 off the swath and 49 within the swath; similar numbers of *C. formicarius* were observed off and within the swath, at 1.88 ± 0.81 and 0.63 ± 0.26 (mean ± se), respectively (Figure 5b; *F*_1,81_ = 3.400, *p* > 0.05). For *E. postfasciatus*, similar numbers were detected off and within the swath, at 0.59 ± 0.47 and 0.41 ± 0.39, respectively (*F*_1,81_ = 0.301, *p* > 0.05).

On 17 October 2020, before the cyantraniliprole application, 30 plants were collected from the field, revealing the presence of 2.93 ± 0.92 *C. formicarius* and 2.17 ± 0.55 *E. postfasciatus* per plant (Figure 5c). On 13 December 2020, 30 plants were collected from the untreated area, and 10 plants were collected from the cyantraniliprole backpack-sprayed area. The mean occurrences (±se) of the two weevil species were 18.50 ± 3.79 and 4.52 ± 1.83 in the untreated area and 4.80 ± 1.63 and 0.20 ± 1.83 in the backpack-sprayed area. On 13 December 2020, 30 plants were collected from the insecticide-free area next to the drone application area, 6 were collected off the swath, and 24 were collected within the swath; the respective per plant mean occurrences (±se) of *C. formicarius* were 6.07 ± 0.88, 7.17 ± 1.12, and 2.17 ± 1.12, while for *E. postfasciatus* they were 6.83 ± 1.66, 9.17 ± 1.09, and 1.75 ± 1.28. The square-root transformed numbers of these two species varied significantly among the locations (*F*_2,57_ = 7.494, *p* < 0.001 and *F*_2,57_ = 6.619, *p* = 0.003, for *C. formicarius* and *E. postfasciatus*, respectively). For both species, the numbers did not vary significantly between the margin area above which the drone flew but did not spray the insecticide and the off-swath area, whereas their numbers differed significantly between these areas and the in-swath area (Tukey’s test, *p* < 0.05).

### 2.4. Quantification of Insecticide and Occurrence of Weevils

**Figure 6:**
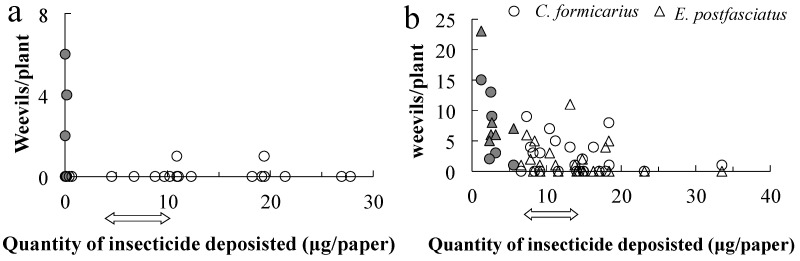
The number of weevils detected in sweet potato plants beside water-sensitive papers in the chlorantraniliprole application in 2020 (**a**) and the cyantraniliprole application in 2020 (**b**). Closed marks indicate the plants off the swath. The two-sided arrows indicate the government-stipulated range of insecticide quantities.

In the chlorantraniliprole experiment in 2020, the total quantity deposited in each event should have been between 4.94 and 9.88 µg per paper on the swath, based on the regulation. The total quantity of insecticide applied was estimated via regression (Figure 3): the quantity of the insecticide deposited was less than the lower limit at all eight off-swath locations (0.20 ± 0.07 µg). Based on the 17 on-swath papers, the mean amount deposited on the swath was 13.97 ± 1.80 µg; the quantity deposited was below the lower limit at five sites, above the upper limit at three sites, and within the stipulated range at nine sites. No *E. postfasciatus* weevils were found on these plants; in contrast, *C. formicarius* was detected on four plants outside the swath (50%) and two within the swath (11.8%), at 2–6 weevils per plant (Figure 6a). The number of weevils observed and the quantity of insecticide applied were significantly negatively correlated (*r* = −0.404, *t* = −2.119, df = 23, and *p* = 0.045).

Cyantraniliprole should have been applied in between 7.62 and 15.26 µg if the drone had sprayed in the registered quantity as indicated by an arrow in Figure 6a. Of the 30 plants collected beside the papers, 6 were off the swath and 24 were on it. Off the swath cyantraniliprole was deposited at 2.93 ± 0.54 µg, while on the swath it was deposited at 13.93 ± 1.18 µg. All of the off-swath papers received less than the lower limit for cyantraniliprole, and the associated plants were infested with one or both species of weevils. Among the on-swath papers, two exhibited less than the estimated lower limit of the pesticide and seven exhibited more than the upper limit, while quantity was within the regulated limits for fifteen. Thirteen plants were infested with *C. formicarius* and twelve were infested with *E. postfasciatus*, while seven plants exhibited no weevils. The number of weevils decreased as the insecticide concentration increased (Figure 6b). Thus, both species were significantly negatively correlated with the estimated quantity (*r* = −0.464, *t* = −2.772, df = 28, *p* = 0.010; *r* = −0.507, *t* = −3.113, df = 28, *p* = 0.004, respectively).

### 2.5. The Drone-Based Pesticide Application on the Grower’s Farm

On 23 September 2020, six days before the insecticide application, sweet potato plants were collected from all of the experimental plots on the grower’s farm to check for pre-experimental differences in the weevil abundance. This revealed no weevils in the untreated control and chlorpyrifos- or chlorantraniliprole-treated plots. No damaged tubers were observed in any of the collected plants. In contrast, 0.73 ± 0.69 *C. formicarius* weevils per plant were detected on the plants collected in the drone application area, and 2.41 ± 2.12% of the tubers per plant were infested with weevils. On 21 November 2020, two months after the insecticide treatments, 2.03 ± 0.82 *C. formicarius* weevils per plant were collected from the untreated fields, and 11.3 ± 4.2% of the tubers per plant were damaged by weevils. No *E. postfasciatus* weevils were collected from any of the plots. The weevil counts and the proportions of weevil-infested tubers differed significantly among the insecticide application modes (*F*_3,112_ = 6.117, *p* < 0.001; *F*3, 112 = 9.849, *p* < 0.001, respectively). Both weevil counts and the proportion of weevil-infested tubers per plant were significantly higher where insecticides were not applied than in the insecticide treatment areas, but they did not differ significantly between the plots where insecticides were applied (*p* < 0.05, Tukey’s test).

## 3. Discussions

Although drone-based spraying involves less time and lower labor costs than other pesticide application methods [1,2,8,28,29,30], it is necessary to examine its pest control efficacy. Here, we assumed that the pesticides should be sprayed evenly within the targeted range in the field. Lighter particles, and especially liquid drops, are more likely to be affected by the driving force created by the drone and wind and thus to drift off target [29,31,32]. Consequently, the drone manufacturer specifies the appropriate wind conditions for drone-based pesticide delivery. If an insecticide is applied using a drone, as per the manufacturer’s specifications, no pesticide should be delivered off the swath [31]. In our study, however, there were crosswinds exceeding 2.0 m/s during all of the experiments, thus potentially violating the manufacturer’s specifications.

The distribution of chlorantraniliprole droplets, as detected using the water-sensitive paper, was asymmetrical in both the 2019 and 2020 experiments. On 16 October 2020, the drone application area near the center of the field received very little insecticide. These inconsistencies may be explained by fact that the dominant wind was northerly in 2019, whereas in 2020 it was southeasterly at the first application and northeasterly at the second application. However, for cyantraniliprole the distribution across or along the swath was not notably unbalanced, probably because of the downward wind from the drone rotors, which may have confined the width of the insecticide distribution [33]. Furthermore, a lower insecticide deposition rate on one side of the flight path would have been compensated for by greater insecticide deposition on the other side [34,35]. Thus, the compensatory effects of the cyantraniliprole application could have been caused by the overlapping of two neighboring flights and the downward wind from the drone.

Sweet potato weevils are more difficult to manage using insecticides than canopy-dwelling insects. The two weevil species examined here are subterranean and rarely emerge from the plant body above the ground or into the aerial space [16,36,37], thus allowing them to escape the influence of insecticides. Hence, their management requires systemic insecticides that can be disseminated differently throughout the plant body depending on the plant growth [37,38,39,40,41,42,43]. Owing to their systemic delivery, cyantraniliprole and chlorantraniliprole are effective against the weevils in sweet potato plant root systems [24,44]. Here, the drone application of these insecticides maintained the weevil populations at levels as low as those achieved via the conventional application. For both species, more weevils were collected from plants that received less insecticide via the drone application. Despite the differences in the quantity of insecticide delivered by the drone in each application, the drone application achieved the same efficacy as conventional application methods. The drone-based chlorantraniliprole application reduced the weevil population, thus promising a satisfactory yield of sweet potatoes.

Although the drone-deposited insecticide droplets were unevenly distributed on the canopy of individual plants, the method delivered the insecticide at dosages within the regulated range, thus effectively reducing the subterranean weevil population. In particular, the repeated paper collections in each 2020 application suggest that the insecticide could be evenly distributed at a precision of 0.30 to 0.40 m^2^. This in turn suggests that even the insecticide distribution within the registered range can be achieved in a 0.54 to 0.63 cm square or a circle of 0.61 to 0.71 m. Therefore, if a plant grew a canopy larger than this size, it could expect to receive enough insecticide in the range of the registered quantity, whereas smaller plants may suffer from insufficient insecticide applications. The estimated minimum size for even drone application should not be a problem in plants with bigger canopy sizes, especially in silviculture which targets trees [45]. However, this size is a concern especially for crops with small canopies such as chili, okra, or beans [46]. In other crops in which canopies grow larger, sufficient insecticide delivery can be expected when their crowns grow over the critical size. For these crops, periodical crown size monitoring is needed for drone imaging to be particularly efficient. Drone sensing is helpful to know the appropriate time for insecticide applications in accordance with the canopy growth [47,48,49,50,51].

The efficacy of these insecticides on these subterranean weevils may be attributed to the insecticides’ systemic delivery. In contrast, non-systemic insecticides may be ineffective at controlling subterranean weevils, even with drone-based applications. Systemic pesticide application may therefore be key to controlling sweet potato weevils. Future research could examine how the benefits of systemic insecticides can be maximized in weevil control via drone applications and should aim to achieve the efficient transportation of the insecticide to hotspots of weevil infestation.

## 4. Materials and Methods

### 4.1. Study Site

The experiments were conducted in southern Japan, during the main season of sweet potato cultivation, in four fields: three on Okinawa Island (24°06′32″ N, 127°40′59″ E) and one on Miyako Island (24°44′28″ N, 125°16′16″ E), both at about 50 m asl. Weather data for the drone flight days (Table S1) were obtained from the Japan Meteorological Agency (https://www.jma.go.jp/jma/indexe.html, assessed on 1 February 2021).

Two diamide insecticides, chlorantraniliprole 5% in Prevason 5 (FMC, Tokyo, Japan) and cyantraniliprole 10.3% in Benevia OD (FMC) (both for spray application, diluted 4000 times, at 1000–3000 L/ha), are registered for the control of lepidopteran insects on sweet potatoes in Japan. However, drone application has been registered only for chlorantraniliprole, at 16 times dilution, at 8–16 L/ha, being equivalent to 25 to 50 g/ha. Here, chlorantraniliprole and cyantraniliprole were diluted 16 times and sprayed at 16 L/ha and 8 L/ha, respectively, using a drone with a 10 L tank (DJI Agras MG-1, Shenzhen, China). The drone specifications are described in Table S2. According to the manufacturer’s instructions, the drone distributes a regulated quantity of insecticide at distances of up to 2 m on each side of the flight path. For comparison, the two insecticides were also applied using a backpack sprayer at 4000 times dilution at 2000 or 1000 L/ha.

### 4.2. Chlorantraniliprole Application by Drone in 2019 and 2020

In 2019, chlorantraniliprole was sprayed onto the fields on Okinawa Island using a drone in order to correlate the occurrence of the two weevil species with the location of the plants in the drone swath. In mid-May, 13 ridges (0.3 m high, 50.0 m long, oriented in an east–west direction) were prepared in a 10 × 50 m sweet potato field. After two weeks, 30 cm slips of the local sweet potato cultivar Churakoi-beni were planted at 0.3 m intervals on each ridge. This variety, commonly cultivated in Okinawa, was easily obtained for the present study. This cultivar is susceptible to sweet potato weevils [18,24]. Dead slips were replaced with new ones, and the replacement was ended on 10 June 2019, which was determined as the planting day.

The field was divided into five plots: two no-insecticide plots, 5.0 m long on both the east and west margins, two 5.0 m water-treated plots inside the no-insecticide plots on both sides of the field, and one drone spray plot, 30.0 m inside the field. On 21 August, 72 days after the planting, 117 water-sensitive papers (76 × 26 mm) were placed above the sweet potato plants on each ridge. Chlorantraniliprole was sprayed using a drone flying at 3 m/s and 2 m above the plant crown. All sweet potato plants on the 13 margins were collected within 2.5 m from the eastern margin in the no-insecticide plots, and all plants were collected from the center of the drone-sprayed plot on 16 September, 5 October, and 16 November (i.e., 98, 117, and 159 d after planting, respectively). These days corresponded to 26, 45, and 87 days after planting, respectively. The collected plants were dissected in order to count all of the sweet potato weevils (*C. formicarius* and *E. postfasciatus*) infesting the plants.

In 2020, using the same field used in 2019, 13 ridges (45.0 m each) were constructed without a control and with a margin of 2.5 m, in both the east and west margins of the fields, to examine whether the chlorantraniliprole spraying pattern would affect weevil occurrence. The field was divided into three plots, as in 2019; the lengths of the two no-insecticide plots on the east and west margins were shortened to 2.5 m, while the size of the drone spray plot remained the same (Figure S1). Immediately before each application, water-sensitive papers were placed on the plant crawl on 16 October 2020 (83 papers) and 27 October 2020 (97 papers). In both applications, an additional 15 papers were set in the same manner. Chlorantraniliprole was then administered by drone. The papers were collected immediately after drone spraying to determine the stained area of each paper from an image of the paper [52]. The additional 30 papers applied in 2020 were used by the QSAI Analysis and Research Center Co., Ltd. (Munakata, Fukuoka, Japan) to independently quantify the insecticide coverage.

The size of the area required to stabilize the insecticide quantity in the range of the registered dose at each application date was estimated by the bootstrap method, in which papers under the drone flight swath were picked up one thousand times randomly. The number of papers to pick up varied from one to one thousand by one integer step. Then, the quantity was summed for each paper number, and the per paper quantity was compared among the summed numbers. The paper numbers that were closed to an apparent asymptote were taken as the papers needed to stably distribute the insecticide within the registration range, and we examined whether the per paper quantity would be confined within the registration. This method was also used for the cyantraniliprole.

### 4.3. Cyantraniliprole Application by Drone

For this experiment, the sweet potato cultivar Tamayutaka, rather than Churakoi-beni, was used because a sufficient number of Churakoi-beni slips were not available. This variety is also susceptible to sweet potato weevils (pers. obs.). Slips of this cultivar were planted on 16 ridges (0.8 m interval, 33.0 m long) in a south–north direction, in a field that was 12.0 m east–west and 33.0 m north–south, on 27 April 2020. The northern marginal area (6.6 m long) was set as the no-drone-spraying area. From the no-drone-spraying zone, 30 plants were randomly selected. These plants were collected on 17 October 2020 to confirm the infestation of two weevil species, *C. formicarius* and *E. postfasciatus*, in the field. The collection revealed weevil numbers (mean ± se) of 2.93 ± 0.92 *C. formicarius* and 2.17 ± 0.55 *E. postfasciatus* per plant. These methods were used to evaluate the treatment efficacy. Subsequently, four plots, each 1.5 m long and 4.0 m wide with five ridges, were established in the no-drone-spraying area, and the following treatments were randomly assigned to these plots: no insecticide treatment for three plots as the untreated controls and backpack sprayer application of cyantraniliprole (diluted 4000 times, at 1000 L/ha), on 28 October 2020, as the conventional chemical management plot.

Also on 28 October 2020, cyantraniliprole was sprayed from the drone onto the remaining area of the field (along a 26.4 m flight path) (Figure S2). The drone traveled over ridges 1, 6, and 11 (counting from the west margin), spraying over 13 ridges, while leaving the 2 eastern ridges insecticide-free. Just before the drone application, seven water-sensitive papers were placed on each of the 12 ridges on the eastern margin. Twelve papers, placed on the center of each ridge, were used to measure droplet coverage and thus to quantify the insecticide dose applied, while the other seventy-two papers were examined only for insecticide droplet patterns. After the application, the field was left until 13 December 2020, when ten plants were collected from the southernmost, central, and northernmost locations where the papers had been laid across the drone flight path. On 13 December 2020, ten plants were randomly selected and collected from each of the four plots at the northern margin.

### 4.4. The Field Trial on a Grower’s Farm

A rectangular sweet potato field measuring approximately 3300 m^2^ was selected on Miyako Island. Slips of the Churakoi-Beni cultivar, obtained from nearby farms, were planted between 22 and 27 May 2020. Fipronil (sold as Prince Bate in the Japanese market; BASF Japan, Tokyo, Japan) was applied at 60 kg/ha by the grower immediately before planting to eliminate pre-existing weevils. Each planting line was separated by 0.8 m, and slips were planted at intervals of 0.3 m on each line. The field was divided into two parts, one on the south margin, 9.9 m long, and the rest approximately 80.0 m long, where chlorantraniliprole was applied by the drone. Three quadrats that were 9.9 m long and 5.0 m wide were set on the south margin, each consisting of six 9.9 m lines, and each quadrat was divided into three 3.3 m long plots of the same size with 60 plants in each quadrat. On 23 September 2020, 10 randomly selected plants from each plot were collected to count the number of infesting weevils. The roots were sorted into two classes, those with tubers weighing ≥50 g and the remainder. The tubers were individually weighed and then dissected to count the numbers of infesting weevils.

On 28 September 2020, an 80.0 m line was randomly drawn in the drone application area, with 30 plants at the center, and both ends were examined for weevil infestation. On 29 September 2020, three treatments were randomly assigned to three quadrats: an untreated control with no insecticide application, chlorpyriphos application around the main stem on the ground surface (in granular formulation, equivalent to 60 kg/ha), and backpack spraying of chlorantraniliprole, diluted 4000 times, at 2000 L/ha. Chlorantraniliprole (16 times diluted, at 16 L/ha) was also applied to the remaining area using drones. During drone application, plants in the other treatment plots were covered with plastic sheets to avoid insecticide drift.

On 22 November, 30 randomly selected plants from each quadrat were collected and examined for weevil infestation. An 80.0 m line was drawn randomly in the drone application area, and 10 plants were selected at the center and both terminal sides to examine weevil infestation.

### 4.5. Data Collection and Analyses

The effects of insecticide treatment on weevil infestation were examined using analysis of variance (ANOVA). To approximate the data distribution to a normal distribution, 0.5 was added to the weevil numbers, which were then square root-transformed, while the proportions of weevil-infested tubers were arcsine-transformed following square root transformation. Tukey’s honest significant difference (HSD) test was performed to compare the means between treatments, and differences were considered significant at *p* < 0.05. The means and standard errors (se) of the non-transformed data are presented. Statistical analyses were performed using R 4.0.3.

## 5. Conclusions

Drone spraying deposited the insecticide droplets unevenly on the canopy of individual plants, whereas the average quantity of the insecticide was within the registered range for the field. Drone praying effectively reduced the populations of the subterranean weevils. The efficacy of insecticides in controlling subterranean insects seems to be due to the systemic delivery of the insecticides in the plants. However, the amount of insecticide reaching the tubers was not measured and remains to be examined in future.

## Data Availability

The regulations of the institute prevent the sharing of the raw data.

## Supplementary Materials

The following supporting information can be downloaded at https://www.mdpi.com/article/10.3390/plants14223511/s1: Table S1. Weather conditions during the five insecticide applications by drone; Table S2. Specifics of the drone used for insecticide application in this study; Figure S1. Chlorantraniliprole distribution in a sweet potato field. The chlorantraniliprole was applied by drone and detected using water-sensitive paper; Figure S2. Cyantraniliprole distribution in a sweet potato field. The chlorantraniliprole was applied by drone and detected using water-sensitive paper.
