# Supplementary material for: Drone Delivery of Insecticide Is Uneven Yet Sufficiently Controls Subterranean Weevils Infesting Sweet Potato Plants
Source: Plants (Basel). 2025 Nov 18;14(22):3511. doi: 10.3390/plants14223511 (PMC12656145; doi:10.3390/plants14223511)
Supplement: Supplementary file 1 [file plants-14-03511-s001.zip › plants-3758761-supplementary.pdf]

plants

MDPI

Table S1. Weather conditions[1] during the five insecticide applications by drone.

Date	Temperature (°C)	Wind velocity (m/sec)	Dominant wind direction
August 21, 2019	29.3–32.5	4.9–6.7	SE
August 26, 2020	29.7–31.9	3.6–4.9	E–ESE
August 27, 2020	27.8–28.4	5.3–7.7	S
August 28, 2020	27.5–31.1	3.0–6.0	ESE –ES
September 29, 2020	26.0–28.2	3.5–4.9	N

[1] In all experiments, it was fine and no precipitation was recorded.
Data from the observatory of the Japan Meteorological Agency, approximately 3 km from the two fields, are provided here.

Table S2. Specifics of the drone used for insecticide application in this study.

Specifics	Performance
Flight velocity	2 m/sec
Flight height	3 m
Flight direction	Parallel to rows
Flight path	Automatically set
Flight capacity	30 min
Rotor diameter	1.52 m
No. of rotors	8
No. of rotors	8
Tank capacity	16 L
Spray rate	0.525 L/min
Spray swath	4 m

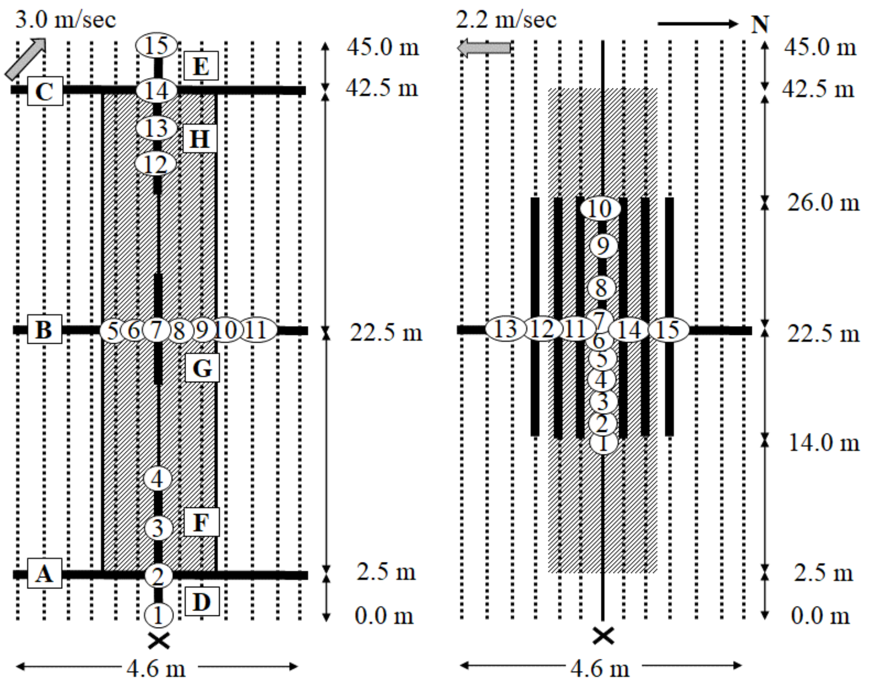

Figure S1. The sweet potato field applied with chlorantraniliprole by the drone (DJI Agras MG-1) on October 16 (left) and 26 (right), 2020. Solid or dotted lines indicate the top of ridges on which sweet potato plants existed in 0.5-m spacing. The drone flew 3.0 m above the solid line with insecticide application from 2.5 to 42.5 m at a speed of 3.0 m/sec. The hatched area indicates both two 2-m sides of the drone flight where insecticide was assumed to be applied in appropriate doses for the weevil control. Thick lines indicate the locations where water-sensitive spray papers were placed beside the sweet potato plants for the calculation of droplets sprayed. Numerals in circle correspond to the papers that were analyzed for the quantification of chlorantraniliprole sprayed. Characters A to G in squares indicate the groups of plant positions according to the spray conditions. These plants were collected for the evaluation of treatment efficacy based on the occurrences of infecting weevils. N with an arrow indicates north direction. Grey arrows indicate the wind direction with the mean speed during the application. The cross at the left bottom indicates the origin of the location of the field, defined as 0 m both on north-south and on east-west.

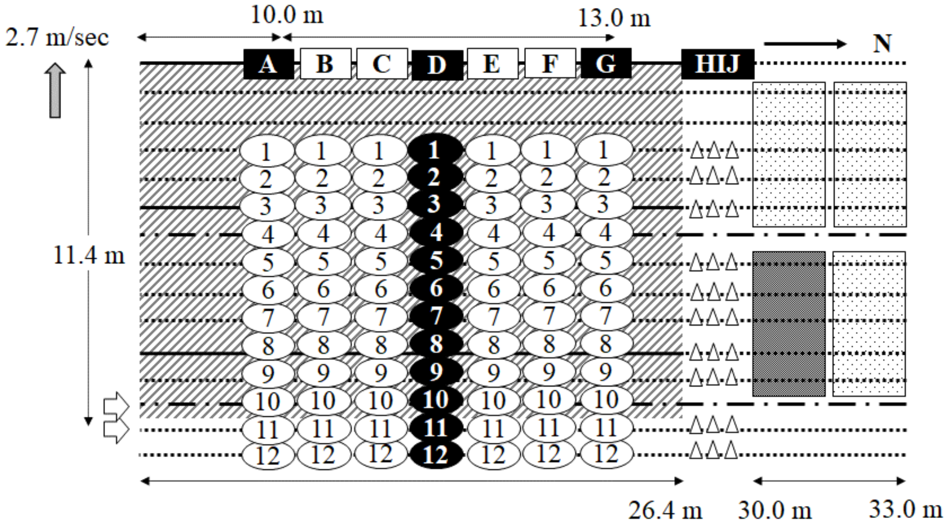

Figure S2. The sweet potato field applied with cyantraniliprole by the drone (DJI Agras MG-1) on October 25, 2020. Solid or dotted lines indicate the top of ridges on which sweet potato plants existed in 0.5-m spacing. The drone flew 3.0 m above the three solid lines 0 to 26.4 m with insecticide application from the south edge at a speed of 3.0 m/sec. The area where the insecticide was assumed to be applied in the determined quantity is hatched. Characters in squares indicate the location across the planting rows. Numerals in black circles indicate the locations where papers were used to quantify cyantraniliprole sprayed. The thick arrow at the top right indicates north direction. Grey arrows indicate the wind direction with the mean speed during the application. Four small plots on the northern edge were those where untreated check (dotted) and cyantraniliprole application by a backpack sprayer (shaded) were randomly assigned. The chain lines indicate the location where no sweet potato plants had been planted. Plants at A, D, and G were collected for weevil infection testing. Plants at H to J, where the insecticide was not sprayed, were collected for weevil counts as untreated check.
